# The effect of gestational diabetes mellitus on sufentanil consumption after cesarean section: a prospective cohort study

**DOI:** 10.1186/s12871-019-0925-1

**Published:** 2020-01-09

**Authors:** Chen Yang, Wei Lian Geng, Jianying Hu, Shaoqiang Huang

**Affiliations:** Department of Anesthesiology, Obstetrics & Gynecology Hospital, Fudan University, 128# Shenyang road, Shanghai, 200090 China

**Keywords:** Gestational diabetes mellitus, Postoperative analgesia, Cesarean section

## Abstract

**Background:**

Previous studies have shown that patients with long-term diabetes require more opioids after surgery than patients without diabetes. Gestational diabetes mellitus (GDM) normally only lasts for a brief period; nevertheless, its effect on sufentanil consumption after cesarean section is unknown.

**Methods:**

This prospective cohort study included two groups: a GDM group (*n* = 32) and a matched non-GDM (NGDM) group (*n* = 32). All patients underwent routine combined spinal-epidural anesthesia for cesarean delivery. Sufentanil consumption through an intravenous patient-controlled analgesia (PCA) pump, the frequency of PCA requests, and visual analog scale (VAS) scores 6 and 24 h after surgery were compared between groups.

**Results:**

Sufentanil consumption (μg) 6 h after surgery was higher in the GDM group than in the NGDM group (24.0 ± 6.6 vs 20.1 ± 5.7, *P* = 0.023). PCA was used more frequently 6 and 24 h after surgery by the GDM group than by the NGDM group (1[0–2] vs 0[0–1], *P* = 0.001; 6 [1–5] vs 3 [1, 2, 6–8], *P* = 0.001, respectively). The VAS score during activity 24 h after surgery was higher in the GDM group than in the NGDM group (5 [2, 3] vs 5 [1, 2], respectively, *P* = 0.03).

**Conclusion:**

Pregnant women with GDM require more opioids during the immediate postoperative period after cesarean section than those without GDM.

**Clinical trials registration:**

No. ChiCTR1800016014, ChenYang, May 6th 2018.

## Background

Gestational diabetes mellitus (GDM) is defined as high blood glucose caused by impaired glucose tolerance detected and diagnosed during pregnancy. GDM is characterized by elevated fasting blood glucose and impaired glucose tolerance during pregnancy. The reference values used in the oral glucose tolerance test for pregnant women are as follows: fasting, 5.6 mmol/L; 1-h postprandial, 10.3 mmol/L; 2-h postprandial, 8.6 mmol/L; 3-h postprandial, 6.7 mmol/L. GDM is diagnosed when two or more test values reach or exceed the reference values. In most cases, GDM resolves 1 to 2 months after delivery (“transient diabetes”). GDM is one of the most common complications of pregnancy, with a prevalence of approximately 3 to 7% [6–8].

Karci et al. showed that the analgesic effect of morphine is negatively affected by high blood glucose in patients scheduled for elective total abdominal hysterectomy. According to data on postoperative intravenous patient-controlled analgesia (PCA), patients with diabetes require more opioids than patients without diabetes [1]. Glycated hemoglobin (HbA1c) is a highly reliable indicator of glycemic control over the previous 8 to 12 weeks [2]. Kim et al. conducted a prospective observational study and found that perioperative HbA1c was positively correlated with postoperative opioid (fentanyl) consumption among patients with diabetes undergoing open nephrectomy [3]. A retrospective cohort study conducted by Weiner et al. showed that total opioid consumption during the 4-month period following surgery was increased in diabetics with operative ankle fractures [4]. However, the mechanism that underlies this relationship is unknown. Chronic high blood glucose might affect opioid receptors, thereby altering the pharmacokinetics and pharmacodynamics of opioids [5]. Alternatively, chronic high blood glucose might affect a patient’s metabolism [9] or neurotransmitter levels [10–12].

The patients included in previous studies had been diagnosed with diabetes for at least 60 weeks [3]; however, the course of gestational diabetes is usually shorter than this, and its association with opioid consumption is unclear. Therefore, we conducted a prospective observational cohort study in which we investigated the correlation between gestational diabetes and sufentanil consumption during the immediate postoperative period after cesarean section. We hypothesized that women with GDM require more opioids during the immediate postoperative period after cesarean section than those without GDM.

## Methods

### Participants

**Fig. 1:**
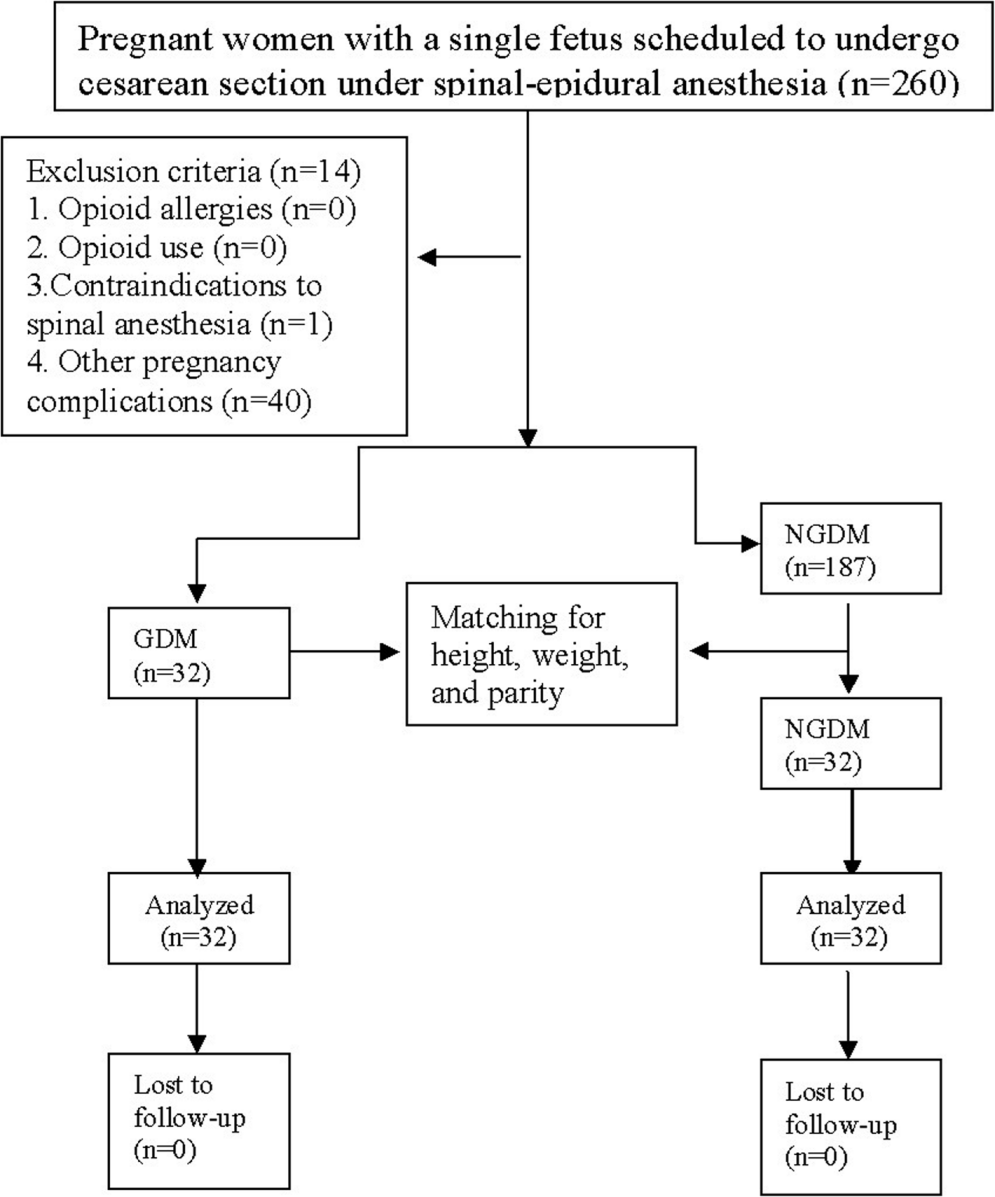
Flow chart for patient enrolment

The Ethics Committee of the Obstetrics and Gynecology Hospital, Fudan University, approved this prospective, observational cohort study, and it was registered at the Clinical Trials Registry (http://www.chictr.org.cn/, Registration No. ChiCTR1800016014). This study was conducted between June 2018 and October 2018 at the Obstetrics and Gynecology Hospital, Fudan University. Inclusion criterion: pregnant women, American Society of Anesthesiology (ASA) II with a single fetus scheduled to undergo elective cesarean section under combined spinal-epidural anesthesia. Exclusion criteria: history of opioid allergies, history of opioid use within the previous week, contraindications for spinal anesthesia, known DM or other pregnancy complications (e.g., gestational hypertension, pregnancy complicated with hypothyroidism, and preeclampsia). Pregnant women with GDM who met the above criteria were included in the GDM group. For each pregnant woman included in the GDM group, a pregnant woman without GDM who matched with respect to height ± 2 cm, weight ± 1 kg, and the same parity was included in the non-GDM (NGDM) group (Fig. 1). Patients signed an informed consent document prior to participation in the study.

### Procedure

On the morning of cesarean section, blood was drawn for measurement of maternal glucose and HbA1c. Maternal age, height, weight, gestational age, and parity data were recorded. In addition, the blood glucose management methods used by patients in the GDM group, such as diet restrictions, oral medications (and doses), and insulin injections (and doses), were recorded.

No medication was given before the operation. In the operating room, an 18-gauge needle was used to puncture a vein in the right upper arm, and an indwelling catheter was placed. Blood pressure, electrocardiograms (ECGs), heart rate, and pulse oximetry were routinely monitored noninvasively. Baseline values were recorded. At the beginning of anesthesia, 6% hydroxyethyl starch was infused at 20 mL/min until delivery. The infusion speed was then adjusted by the anesthesiologist according to the maternal circulation state until the total amount of 500 ml hydroxyethyl starch had been infused and replaced with Ringer’s lactate solution. A combined spinal-epidural anesthesia was performed at the L3–4 or L2–3 vertebral interspace with the patient in the left lateral position. An 18-gauge Tuohy needle was placed in the epidural space and advanced until there was loss of resistance to saline; then, a 25-gauge Whitacre spinal needle was inserted through the Tuohy needle until the dura mater was punctured. Next, 8~10 mg bupivacaine was diluted to 3 ml with cerebrospinal fluid for intrathecal injection, and an epidural catheter (3–4 cm) was immediately placed. The patient was placed in the supine position, and the operating table was tilted to the left. During the first 10 min after spinal anesthesia administration, a needle was used to test the sensory block level every 2 min. The operation began when the block level reached T6. Patients who did not achieve this level were excluded from the study, and 1.5% lidocaine was injected epidurally until successful anesthesia was achieved. During the operation, the patient received continuous supplementary oxygen through a mask at 5 L/min. If hypotension occurred (i.e., systolic blood pressure < 90 mmHg or a decrease of > 20% from baseline), 40 μg of phenylephrine was given intravenously (iv) and repeated as needed; furthermore, the infusion rate of hydroxyethyl starch was increased. If sinus bradycardia occurred (heart rate < 50 bpm), 0.2 mg of atropine was given iv, and this was repeated as needed. If pain occurred after delivery but during the operation, it was treated with intravenous analgesics.

After the infant was delivered and the umbilical cord had been clamped, 50 mg of flurbiprofen and 4 mg of ondansetron were administered intravenously. At the end of the operation, 5 μg of sufentanil (diluted in saline to 5 mL) was given via epidural injection, and the epidural catheter was removed. The operative time and blood loss were recorded.

After the operation, the patient was moved to the post anesthesia care unit (PACU). When the patient’s blood pressure and heart rate were normal and the anesthesia level was T6 or below, an intravenous analgesia pump (Aipeng, Nantong Apon Medical Devices Co., Ltd.) was connected, and the patient was instructed in its proper use. The analgesics given via patient-controlled intravenous analgesia (PCIA) included sufentanil 150 μg and ondansetron 4 mg diluted in saline to 150 mL; the sufentanil concentration was 1 mg/ml. The background dose was 3 mL/h, with a bolus dose of 3 mL and a lock-out time of 15 min. No other postoperative analgesics were administered during the study period. The anesthesia nurse involved in the study recorded the use of the postoperative analgesia pump (sufentanil consumption and number of PCA compressions [reflecting maternal need]) as well as adverse reactions such as nausea, vomiting, and itching. Patients with nausea and vomiting were given ondansetron (4 mg, iv), which was repeated as needed. Moreover, a visual analog scale (VAS, 0 cm ~ 10 cm) was used to assess pain during rest and activity 6 and 24 h after the operation. In addition, patient satisfaction with the postoperative analgesia was evaluated as 1 (very dissatisfied), 2 (dissatisfied), 3 (neither dissatisfied nor satisfied), 4 (satisfied), or 5 (very satisfied). Patients were excluded from this study if general anesthesia or intraoperative intravenous opioids were used, if other medications were administered intraoperatively through the epidural, if they underwent hysterectomy due to bleeding or for other reasons, if they discontinued the analgesia pump for any reason (e.g., severe surgical or medical complications), or if they asked to be withdrawn from the study early.

### Statistical analysis

The primary endpoint was sufentanil consumption 6 h after the operation. The secondary endpoints included sufentanil consumption 24 h after the operation, the frequency of PCA press 6 and 24 h after the operation, the VAS score, adverse reactions during postoperative analgesia administration, and patient satisfaction with postoperative analgesia. The Kolmogorov-Smirnov test was performed to determine whether the data displayed a normal distribution. Normally distributed measurement data are expressed as the mean ± standard deviation and analyzed using an independent-samples t-test. Categorical data (e.g., nausea, vomiting, and itching) were analyzed using Fisher’s exact test. SPSS v22.0 (SPSS Inc., Chicago, IL, USA) was used for all analyses, and *P* < 0.05 was considered significant. A multivariate analysis of covariance was performed to eliminate the offset effect of the statistically significant variables in the two groups on the results.

The primary endpoint was sufentanil consumption 6 h after the operation. We referenced a previous study that used the same analgesia regimen used in this study to estimate the necessary sample size [13]; in that study, NGDM patients used 15.8 ± 6.3 μg of sufentanil 6 h after the operation. We determined that the difference between GDM patients and NGDM patients must be at least 20% (4.6 μg) to reach clinical significance. With two-tailed tests, α = 0.05 and β = 0.2, and at least 29 patients were required in each group. Ultimately, 32 patients were included in each group, allowing for a 10% attrition rate.

## Results

All patients (32 in each group) completed this study (Fig. 1). The demographic and intraoperative characteristics of the patients are shown in Table 1. The women in the GDM group were older and had a lower mean gestational time than those in the NGDM group.

**Table 1:** Clinical characteristics of the patients

	GDM (*n* = 32)	NGDM (*n* = 32)	*P*
Age (years)	34.3 ± 0.1	32.1 ± 0.1	0.04
Gestation (weeks)	37.9 ± 1.2	38.8 ± 1.2	0.004
Height (cm)	162.1 ± 3.2	160.9 ± 4.0	0.24
Weight (kg)	69.3 ± 8.5	69.8 ± 8.6	0.48
BMI (kg/m^2^)	26.1 ± 3.3	26.9 ± 3.1	0.24
Parity (first/repeat)	14/18	14/18	1
Amount of bleeding (ml)	310 ± 74	315 ± 70	0.34
Duration of surgery (min)	45. 3 ± 4.2	46.5 ± 4.7	0.32
Blood glucose (mmol/l)	4.8 ± 0.2	4.3 ± 0.4	0.0001
HbA1c (%)	5.9 ± 1.9	4.9 ± 0.3	0.003
Newborn weight(g)	3146 ± 214.8	3296 ± 149.2	0.21

Data are presented as the mean ± SD or number

Preoperative laboratory tests showed that fasting blood glucose (mmol/l) was higher in the GDM group than in the NGDM group, despite blood glucose management in the GDM group (diet restriction, medication, and insulin). HbA1c was significantly higher in the GDM group than in the NGDM group. No significant between-group differences were observed with regard to other indicators.

The data on analgesic use are shown in Table 2. The GDM group used the analgesia pump more frequently than did the NGDM group 6 and 24 h after the operation (*P* < 0.005). Sufentanil consumption (μg) 6 h after the operation was higher in the GDM group than in the NGDM group (24.0 ± 6.6 vs. 20.1 ± 5.7, *P* = 0.023). Sufentanil consumption 24 h after the operation in the GDM group and the NGDM group did not differ significantly.

**Table 2:** Postoperative analgesia

	Number of PCA compression		Sufentanil consumption (ug)		VAS scores (cm)			
					Rest		Movement	
	6 h	24 h	6 h	24 h	6 h	24 h	6 h	24 h
GDM *n* = 32	1[0–2]	6[5–8]	24.0 ± 6.6	85.4 ± 12.4	2[1.5–3]	2[1–2]	2.5[1–3]	5[5–6]
NGDM *n* = 32	0[0–1]*	3[1–5]*	20.1 ± 5.7^#^	80.5 ± 13.5	2.5[2–3]	2[2–3]	2.5[2–3]	5[4–5]^#^

Data are presented as the mean ± SD or median [IQR]. *P < 0.005 vs GDM; ^#^P < 0.05 vs GDM

The results of multivariate analysis of covariance showed that age (*F* = 0.893, *P* = 0.348) and gestational week (*F* = 0.005, *P* = 0.944) had no significant effect on opioid consumption 6 h after surgery. After controlling for age and gestational week, opioid consumption 6 h after surgery was still significantly higher in the GDM group of pregnant women (24.2 μg, 95% CI: 21.7–26.6 μg) than in the NGDM group (20.1 μg, 95% CI: 17.6–22.5 μg) (*F* = 5.097, *P* = 0.028). The difference in opioid consumption between the groups was 4.1 μg (95% CI: 0.5–7.7 μg) (Table 3).

**Table 3:** Covariance analysis of sufentanil consumption 6 h after surgery between two groups

Source	Sum of Squares (Class III)	Degrees of Freedom	Square of Average	F-value	*P*-value
Modified model	261.558	3	87.186	1.998	0.124
Intercept	31.749	1	31.749	0.728	0.397
Age	38.968	1	38.968	0.893	0.348
Gestational week	0.216	1	0.216	0.005	0.944
Group	222.408	1	222.408	5.097	0.028
Error	2618.176	60	43.636		

The VAS scores during rest and activity were assessed 6 and 24 h after the operation (Table 2). The VAS score during activity 24 h after the operation was higher for the GDM group than for the NGDM group (5 [2, 3] vs 5 [1, 2], *P* = 0.03). No significant between-group differences were found in the pain scores at the 6-h postoperative rest/activity measurement or at the 24-h postoperative rest measurement.

No significant between-group differences were observed in adverse reactions during postoperative analgesia administration or with regard to patient satisfaction with postoperative analgesia (Table 4).

**Table 4:** Comparison of adverse reactions and patient satisfaction between two groups

	GDM (*n* = 32)	NGDM (*n* = 32)
Nausea	5(16)	4(13)
Vomiting	1(3)	0(0)
Pruritus	3(9)	1(3)
Satisfaction (1/2/3/4/5)	0/0/3/27/2	0/1/1/29/1

Data are number (%)

## Discussion

This prospective cohort study showed that pregnant women with GDM require more opioids and exhibit higher sufentanil consumption 6 h after cesarean section than NGDM patients.

In animal models of diabetes mellitus (DM), high blood glucose has been shown to reduce the effectiveness of opioid receptor agonists [14–16]. Studies on relevant mechanisms have shown that high blood glucose is associated with changes in the expression of opioid receptor genes [17, 18], the body’s metabolism [9], and neurotransmitter levels [11, 12].

Previous studies were conducted in patients who had displayed elevated blood glucose (diabetes) for more than 60 weeks [1, 3]. In contrast, this study was conducted among pregnant women who were diagnosed with GDM after an abnormal glucose tolerance test at approximately 24 gestational weeks, and women with pre-GDM were excluded. As a result, the course of high blood glucose was only approximately 100 days, far briefer than that in a typical patient with diabetes. Nevertheless, this study showed that GDM patients consumed significantly more opioids 6 h after surgery than NGDM patients. The results are similar to those of previous studies of DM patients [1, 3]. This finding is important because few previous studies have focused on GDM, which represents a particular group of hyperglycemic women. Our study shows that GDM has the same positively correlated as diabetes on the demand for opioid analgesics despite the brief period of elevated blood glucose.

Kim et al. showed that preoperative HbA1c is positively correlated with the postoperative opioid needs of patients with diabetes [3]. In our study, the patients in the GDM group were subgrouped based on whether their HbA1c was > 6%, given that the normal upper limit of HbA1c is 6%. However, we found no significant between-group differences in postoperative analgesia consumption, which was not showed in the results, most likely because the sample size was small (HbA1c was > 6% in only six GDM patients). Additional studies are needed to validate this hypothesis.

Notably, this study showed a significant between-group difference in the frequency of PCA use, indicating that the GDM group required significantly more postoperative analgesia than the NGDM group. Moreover, sufentanil consumption was significantly higher in the GDM group than in the NGDM group 6 h after surgery, with no significant between-group difference in VAS scores. Although sufentanil consumption did not differ significantly between the two groups at 24 h, the significantly higher VAS scores and greater PCA use, which reflect maternal needs, suggest an increased opioid requirement in the GDM group that was limited by the “lock-out time” setting of the analgesic pump.

This cohort study matched conditions between the two groups with regard to factors such as weight and height that affect the demand for analgesics. Furthermore, previous studies have shown that contraction pain is more severe after a repeat cesarean section than after a first cesarean section [19]; therefore, parity was also used as a matching condition. Maternal age and gestational age were not matched. On the one hand, advanced age is a high-risk factor for GDM, and the gestational termination times at which pregnant women with GDM undergo cesarean section are earlier. On the other hand, no studies have indicated that maternal age or gestational week influences the demand for analgesics. Our results confirmed these suggestions, and a covariance analysis of the two variables showed that maternal age and gestational age did not affect the results.

This study has some limitations. First, GDM is diagnosed when two or more test values in the oral glucose tolerance test reach or exceed the reference value. The diagnostic criteria were strictly followed, and the patients were instructed to fast for 8 to 12 h prior to the glucose tolerance test. However, some patients might not have followed this instruction, leading to false positive results. We did not ask the patients to undergo retesting, although the inclusion of these patients would only reduce between-group differences. Second, this study only addressed analgesia by intravenous sufentanil after cesarean section. However, many hospitals routinely use intrathecal morphine and intravenous NSAIDs for postoperative analgesia. Whether GDM affects the analgesic effect of the latter drugs remains to be further studied. In addition, because of the design of the cohort study, a double-blind condition was not possible; however, the researchers responsible for the follow-up assessment were unaware of the study groups to which individual patients belonged, and the primary outcome was the objective analgesic pump data; thus, the objectivity of the research results was not affected. Finally, we recorded patient analgesic consumption up to 24 h after surgery; we did not investigate later time points because we usually prescribe 24-h administration of routine intravenous analgesia given that patients typically start eating after 24 h and can take oral analgesics as needed. Moreover, milk production is usually low (mean: < 10 mL/d) during the first 2 days after surgery and then begins to increase. Thus, the short-term use of regular doses of intravenous opioids after delivery results in extremely low drug concentrations in colostrum, with negligible drug intake among infants [20]. Based on these findings, 24-h postoperative intravenous analgesia is safe for nursing infants and helps mothers resume activities as soon as possible after delivery. The results of this study are only applicable to the early postoperative period after cesarean section; whether differences in postoperative long-term analgesia use also occur should be explored in future studies.

## Conclusion

Pregnant women with GDM require more analgesics and exhibit higher sufentanil consumption during the immediate postoperative period after cesarean section than NGDM patients. Clinicians should focus on postoperative analgesia management in GDM patients to improve the effectiveness of postoperative analgesia and increase patient satisfaction.

## Data Availability

The datasets used and/or analyzed during the current study are available from the corresponding author on reasonable request.

## Abbreviations

ASA: American Society of Anesthesiologists
GDM: Gestational diabetes mellitus
HbA1c: Glycated hemoglobin
NGDM: Non-GDM
PCA: Patient-controlled analgesia
PCIA: Patient-controlled intravenous analgesia
SPSS: Statistical package for social science
VAS: Visual analog scale
